# An ANN models cortical-subcortical interaction during post-stroke recovery of finger dexterity

**DOI:** 10.1088/1741-2552/ad8961

**Published:** 2024-11-15

**Authors:** Ashraf Kadry, Deborah Solomonow-Avnon, Sumner L Norman, Jing Xu, Firas Mawase

**Affiliations:** 1 Faculty of Biomedical Engineering, Technion—Israel Institute of Technology, Haifa, Israel; 2 Biology and Biological Engineering, California Institute of Technology, Pasadena, CA, United States of America; 3 Department of Kinesiology, University of Georgia, Athens, GA, United States of America; 4 Equally contributed.

**Keywords:** motor control, finger dexterity, stroke, motor recovery

## Abstract

**Objective.:**

Finger dexterity, and finger individuation in particular, is crucial for human movement, and disruptions due to brain injury can significantly impact quality of life. Understanding the neurological mechanisms responsible for recovery is vital for effective neurorehabilitation. This study explores the role of two key pathways in finger individuation: the corticospinal (CS) tract from the primary motor cortex and premotor areas, and the subcortical reticulospinal (RS) tract from the brainstem. We aimed to investigate how the cortical-reticular network reorganizes to aid recovery of finger dexterity following lesions in these areas.

**Approach.:**

To provide a potential biologically plausible answer to this question, we developed an artificial neural network (ANN) to model the interaction between a premotor planning layer, a cortical layer with excitatory and inhibitory CS outputs, and RS outputs controlling finger movements. The ANN was trained to simulate normal finger individuation and strength. A simulated stroke was then applied to the CS area, RS area, or both, and the recovery of finger dexterity was analyzed.

**Main results.:**

In the intact model, the ANN demonstrated a near-linear relationship between the forces of instructed and uninstructed fingers, resembling human individuation patterns. Post-stroke simulations revealed that lesions in both CS and RS regions led to increased unintended force in uninstructed fingers, immediate weakening of instructed fingers, improved control during early recovery, and increased neural plasticity. Lesions in the CS region alone significantly impaired individuation, while RS lesions affected strength and to a lesser extent, individuation. The model also predicted the impact of stroke severity on finger individuation, highlighting the combined effects of CS and RS lesions.

**Significance.:**

This model provides insights into the interactive role of cortical and subcortical regions in finger individuation. It suggests that recovery mechanisms involve reorganization of these networks, which may inform neurorehabilitation strategies.

## 1. Introduction

Humans, like other higher mammals, exhibit incredible finger dexterity. The skillful ability to move one or more fingers independently enables a large motor repertoire in mammals with prehensile digits. We rely on the ability to individuate fingers in a variety of daily activities, such as typing, tying shoelaces, or handling utensils and various tools. Thus, any injury or pathology that interferes with finger individuation negatively impacts quality of life. After stroke, most people suffer from distal movement impairment which frequently manifests itself as both a decrease in finger strength and prominent deficit in hand dexterity. This is reflected by increased finger enslaving, or unintended force produced by the uninstructed fingers (i.e. inadequate finger individuation) [1–7]. Although a stroke patient may functionally recover the ability to flex and extend all fingers simultaneously, the finger individuation ability often remains deficient. A longitudinal study that tracked finger individuation in stroke patients throughout the acute, sub-acute, and chronic phases revealed that recovery of finger individuation remains far from the level of healthy individuals and tends to plateau between 3–6 months after the stroke event [5]. Nevertheless, the neural mechanism by which recovery of finger individuation occurs is still unclear.

Previous studies have shown the crucial role of the corticospinal tract (CST), originating from the primary motor cortex (M1) and the premotor cortex, in fractionated finger movements [8–10]. Experimental lesions or injury of the motor cortex or corticospinal (CS) region produce a serious detriment to individuated finger movements, resembling that seen in humans after stroke [8, 11, 12]. Recovery of finger dexterity from cortical lesions also reveals interesting results about the involvement of subcortical regions. In particular, the reticulospinal tract (RST), which originates from the reticular formation in the brainstem, was reported to undergo functional changes by modulating its activity after CS lesions during a fine independent finger movement task [13–15]. Direct lesion of the brainstem medial reticulospinal (RS) region, on the other hand, affected mainly posture, strength, and gross movements, while hand function remained unaffected [16]. In parallel, it seems that the CS and RS both have roles in control of contraction force, though the RS may be directly related to control of gross force production, whereas the CS may relate more to the control of fine movements [17].

These observations suggest the existence of a neural circuit with interactive dynamics between the CS and RS that receives inputs (i.e. movement commands) and produces the necessary motor outputs (i.e. finger movements). Critically, changes in the connectivity at all levels seems to play a pivotal role in shaping recovery of finger movement after a brain lesion [15]. How exactly the cortical-reticular circuit reorganizes and contributes to the recovery process of finger individuation after stroke remains an open question.

We investigate how the cortical-reticular circuit may reorganize by developing a physiologically based computational model that is able to predict healthy behavior of finger movement, as well as behavior during the recovery period early after stroke. To date, most related works in modeling motor recovery have been limited to simulating either only wrist flexion force, or single-finger strength and individuation compared to the rest of the fingers [18, 19]. In particular, Norman *et al* [18] presented a computational neural network model based on a stochastic reinforcement algorithm for a one-finger task and separately simulated the force patterns of the instructed finger (index) and the uninstructed finger (middle) in a non-lesioned normal mode, then independently in a lesioned stroke mode. Simulating the normal condition separately from the stroke condition limits the mechanistic understanding of how the network reorganizes and contributes to the recovery process of finger individuation after stroke. Here we present a complete solution that stems from a single simulation of a network at different conditions. This advance is crucial to better understand the possible neurophysiological mechanisms that might underlie stroke recovery.

In the present study, we built a novel artificial neural network (ANN) model of the hand upper neuromotor system that simultaneously models two fingers, alternating between instructed and uninstructed modes with different force levels. Our ANN model captures residual capacity and dynamics at the cortical, subcortical and behavioral levels of finger recovery following a stroke. Importantly, our solution is complete in that once initialized to the ‘normal’ condition (i.e. prior to stroke), it is capable of simulating the different stages of the cortical motoneurons throughout the stroke event and the recovery process.

Notably, the structure of our ANN followed normal anatomical connectivity constraints to impose physiologically-based structure on known features of cortical and subcortical connectivity [20, 21]. This structure allowed the ANN to seek an optimum over a very broad range of dynamics, not limited by prior knowledge of finger recovery. The model findings also predict that post-stroke CS/RS integrity is correlated with level of finger dexterity recovery; a finding which can be validated in a clinical setting and, if successful, could inform patient treatment.

## 2. Methods

### 2.1. Model description

**Figure 1.**
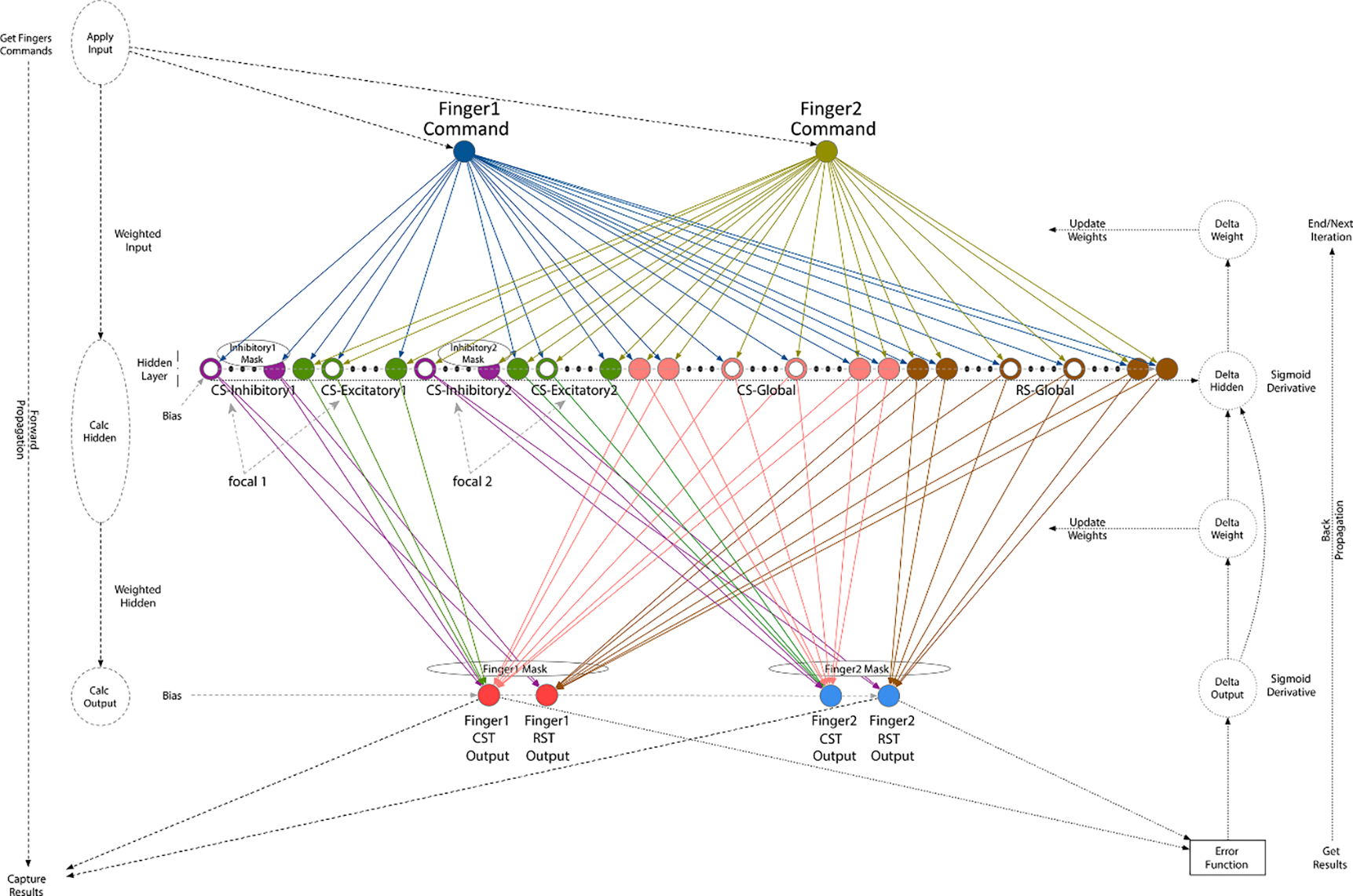
ANN Architecture Diagram. The inputs represent the pre-commands generated in the pre-motor cortex division relative to the two fingers. The hidden layers represent the primary motor cortex division (CS) and other sub-cortical motor regions (RS) involved in the control of voluntary movement. The outputs represent the CST and RST of each finger. Different colors represent different functions and/or a different finger. The neurons are represented by small circles (empty circles = neurons ‘disabled’ by stroke, filled circles = healthy neurons). Dotted circles and lines represent the backpropagation flow. Abbreviations: CS—corticospinal, RS—reticulospinal. Note that inhibitory CS neurons represent excitatory pyramidal tract neurons that project to inhibitory spinal interneurons.

We developed a clustered ANN model constructed from 3 layers: input, hidden and output (see figure 1, ANN Architecture Diagram). The input layer represents the commands for finger movements generated by the pre-motor cortical area. The hidden layer, the computational heart of the model, represents the cortical primary motor neurons and the brainstem reticular neurons in the medulla/pons. The output layer represents the task action outcome of the CST and RST driving the spinal motoneuron pools and muscles.

#### 2.1.1. Input layer

The input layer is composed of two movement command inputs (see section 2.2.1, Mathematical Definition, CMD), one for each of the two fingers, indicating whether each finger is instructed or uninstructed. The desired force level that the instructed finger should apply is encoded within the command value of the instructed finger. There is no force level for the uninstructed finger since it should not apply force, and any force resulting from the simulation is considered enslaving (unintended force production). Four combinations for the command movement inputs can be defined for the two fingers, depending on the desired task (e.g. 100% force): instructed/uninstructed (1, −1), uninstructed/instructed (−1, 1), instructed/instructed (1, 1) and uninstructed/uninstructed (−1, −1). In this study, we focused on the first two commands (i.e. 1/−1 or −1/1) as they demonstrate individuation between the two fingers. The encoded force in the instructed finger command is in the range of (0,1], representing the entire possible force range from 0% to 100% of the maximum force. The value 0 is an illegal value for the instructed finger command. The value −1 for the uninstructed finger command represents zero force.

#### 2.1.2. Hidden layer

The hidden layer is based on a simplified structure of the motor control areas, representing separable motor control functions and organized into four different groups: for fine movement, two dedicated focal CS neuron groups, each including excitatory and inhibitory neurons, and one shared excitatory CS neuron group, and for gross movement, one shared excitatory RS neuron group [13, 22, 23]. Each finger’s neuron cluster is a combination of its dedicated excitatory and inhibitory focality groups and the shared CS and RS groups [13]. The hidden layer state variables (see section 2.2.1, Mathematical Definition, HO) hold the intermediate hidden layer neurons’ output values. Note that the ‘inhibitory neurons’, referred to as such for simplicity, represent excitatory pyramidal neurons that project to inhibitory spinal interneurons, therefore resulting in an inhibitory effect [24, 25].

#### 2.1.3. Output layer

The output layer has four force outputs, two associated with each finger (see section 2.2.1, Mathematical Definition, FO), one for the CST and one for the RST. The expected outputs are the outcomes of the instructed/uninstructed fingers’ commands (see equation (8)).

#### 2.1.4. Input layer to hidden layer connectivity

The fingers’ command inputs (instructed/uninstructed) are connected to all hidden layer neurons [26], with the exception that each command affiliated to one finger does not connect to the inhibitory neuron group of the other finger. Inhibitory neurons constitute 20% of the total neurons in the hidden layer, split between the two fingers [27]. The strength of the connectivity between the layers is described by the connection weights (see section 2.2.1, Mathematical Definition, WH). A focal neuron in our network (either excitatory or inhibitory) may be defined as a neuron that can be driven by multiple neurons, but is able to drive only one downstream neuron, or can be driven by a single neuron while driving multiple downstream neurons. A shared CS or RS neuron is defined as a neuron that can be driven by multiple neurons and is able to drive multiple downstream neurons.

#### 2.1.5. Hidden layer to output layer connectivity

The hidden layer neurons are connected to the output layer neurons (finger outputs). Each finger is associated to two outputs (CST and RST outputs), and the connectivity is based primarily on each finger’s cluster. Each finger’s CST output is driven independently by its cluster of neurons and from the shared CS neurons. The inhibitory neuron groups also drive their affiliated finger’s RST output, while the RS neuron group drives both fingers’ RST outputs. Again, connectivity strength between the layers is represented by the connection weights (see section 2.2.1, Mathematical Definition, WO).

#### 2.1.6. Bias parameters

Bias in neural networks involves a mathematical operation that can be thought of as analogous to the role of a constant in a linear function, whereby the line is effectively translated by the constant value. Both the hidden layer and output layer neurons connect to bias constants. We added these constants to the sum of the inputs to the neurons and used them to shift the input values so that the outputs of the computation functions fit within the desired range of output values. The bias is required when the summed weighted inputs of each neuron require adjustment before applying the activation function and allows the NN model to optimally fit data (see additional details in section 2.2.1, Mathematical Definition, in particular BH and BO).

### 2.2. Model definition

The ANN model is characterized by three key features. (1) The number of residual neurons in the hidden layer is inversely proportional to the magnitude of the lesion overlap with the CS region and the deficit (i.e. ‘dead’ neurons) in the RS region. The degree of residual motor function is highly dependent on lesion load in the CS region (i.e. CST integrity), but not necessarily on lesion size per se [28–32]. (2) The force that each finger muscle generates is determined by the weighted sum activities of cortical and sub-cortical neurons in the hidden layers. Muscle force production is typically proportional to the firing rate of neurons in the motor cortex [17, 33]. We therefore assumed that increase in the firing rate of a single neuron caused proportional increase in muscle force, up to a saturation limit, with the proportionality constant determined by the connection weights. (3) Lastly, we assumed that the motor system must find this solution by evaluating the results of task performance based on the deviation of the net force output of the fingers from the desired force targets (i.e. error function as teaching signal).

This type of error-based learning uses summary feedback of motor performance to update synaptic weights and can be achieved with computations implemented locally at synapses and is thus considered biologically plausible. We perform feedforward-propagation followed by a backpropagation iterations algorithm to optimize for the results convergence. We compare the task performance in each feedforward pass and the error function is minimized during the back-propagation pass. The iterations are repeated until reaching or approaching the global minimum of the error function.

#### 2.2.1. Mathematical definition

The fingers’ command input variables, ${CMD}_{i;i=1:NI\in \{2,3,4,5\}}$, (see section 2.2.2, Initialization and Parameter Setting, NI=2, Finger 1 Command and Finger 2 Command in figure 1) are used to capture a semi-binary-type (instructed/uninstructed) movement task that is required from each finger. The command’s binary movement information is encoded as +1/−1 rather than 1/0 to represent instructed/uninstructed movement tasks (a value of 0 does not work well with the ANN inputs, as all computation results would be zero regardless of its weights). The force parameter is defined as a number in the range of (0,1], indicating a percentage of full force, and encoded into the instructed finger command (a value of 0 is not allowed, as movement cannot be achieved with zero force, a value of 1 represents 100% of the force). The force parameter, FRC, is associated with the instructed finger (or fingers) based on the command input variables.

The commands, ${CMD}_{i}$, are weighted by the weight links connecting the inputs to the hidden layer neurons: ${WH}_{ij;i=1:2,j=1:N}$, (see section 2.2.2, Initialization and Parameter Setting, N=400). The weighted commands, ${\left({CMD}_{i}\times {WH}_{ij}\right)}_{i=1:2,j=1:400}$, transform to values in the open interval (−1, 1). The appropriate weighted inputs are summed to values in the range of (-∞,+∞) and fed to a corresponding activation function of each hidden layer neuron. We use the sigmoid function,

(1) $$f(x)=\frac{1}{\left(1\;+\;e^{-x}\right)},$$

as the activation function. Here, the input to the hidden layer activation function,

(2) $$\begin{array}{l} x_{j;j=1:400}=\sum_{i=1}^{2}\;{\left({CMD}_{i}\times {WH}_{ij}\right)}_{j=1:400}\\ \;+\;{BH}_{j;j=1:400,} \end{array}$$

is the sum of the weighted commands that project to any one given hidden layer neuron, plus a corresponding bias, ${BH}_{j;j=1:400}$ (described below), and is in the range of (-∞,+∞), representing the summed firing rates of the neurons. The output values of the function, ranging in (0,1), represent the weighted activity of the neurons.

The biases, ${BH}_{j}$ are used to shift the activation function of the hidden layer neurons and stabilize the learning process. These biases shift the neurons’ sigmoid activation functions (equation (1) to the desired range of values (see section 2.1.6, Bias Parameters). Because the inputs to the hidden layer affiliated with the instructed finger must be positive values (reflecting excitatory activity), without bias the outcome values of the activation function (equation (1)) will most likely be in the upper range of (0,1), e.g. [0.5,1) (sigmoid of x⩾0). The lacking portion, (0,0.5), of the desired range, (0,1), can only be reached when applying the sigmoid function to negative input values. Without bias, the weights ${WH}_{ij}$ would then start to switch to negative values during the learning process of the ANN, contradicting the excitatory context. Thus, the output of the hidden layer activation function is tuned to the range of (0,1) using the bias. The inhibitory neurons are negated using a dedicated hidden layer status variable, ${NS}_{j;j=1:400}$. When ${NS}_{j}=1$, this indicates an excitatory neuron, while if ${NS}_{j}=-1$, it indicates an inhibitory neuron. A lesion is applied using this same status variable. That is, each CS or RS neuron is initialized to 1 or −1 for healthy/active neurons and switched to 0 for ‘dead’ (inactive) neurons due to stroke. The hidden layer neuron outputs are then defined as:

(3) $${HO}_{j;j=1:400}={NS}_{j;j=1:400}\times f.$$

These intermediate outputs, ${HO}_{j}$, are then weighted via the weight links, ${WO}_{jk;j=1:400,k=1:NO}$, (see section 2.2.2, Initialization and Parameter Setting, NO=4, represents the 4 finger outputs: CST and RST for each of the 2 fingers). These weighted outputs, ${\left({HO}_{j}\times {WO}_{jk}\right)}_{j=1:400,k=1:4}$, yield new values in the range (0,1). The sum of the appropriate values, within the range (-∞,+∞), are fed into the corresponding activation function of the output layer neurons, again the sigmoid function (equation (1)). The biases, ${BO}_{k}$, are used to shift the output layer activation function in order to tune the output to the range (0,1) and stabilize the learning process. Despite the existence of the inhibitory neurons that enforce negative values, the summed inputs of the activation functions corresponding to these outputs still require tuning to the desired range. Thus, in general, for any given neuron in the output layer,

(4) $$x_{k;k=1:4}=\sum_{j=1}^{400}\;{\left({HO}_{j}\times {WO}_{jk}\right)}_{k=1:4}+{BO}_{k;k=1:4}.$$

The outputs of the activation function at the output layer represent the force outputs of CST and RST,

(5) $${FO}_{k;k=1:4}=f,$$

for the two fingers as a percentage of relative force of the movement (0—no force, 1—full force). The instructed finger will show actual instructed force, while the uninstructed finger will simultaneously ‘unintentionally’ activate and show the uninstructed (i.e. involuntary) force.

In addition, we defined mask variables to control the interactions between different layers. They enforce connectivity limitations between different groups of neurons, as they represent different motor functions (simplified motor divisions) that do not necessarily directly interact. To model the interaction between the input and hidden layer groups of neurons, inhibitory masks, ${IM}_{ij;i=1,2,j=1:400}$, are defined. Inhibitory neurons of one finger are affected by their associated finger command, but not by the other finger’s command (hence, ${IM}_{1j}$ and ${IM}_{2j}$ reflect this). For the interaction between the hidden layer and finger output layer, finger masks, ${FM}_{jk;j=1:400,k=1:4}$, are defined. Focal neurons (excitatory and inhibitory) for each finger output tract (k=1,2 for Finger 1 CST and RST, respectively; k=3,4 for Finger 2 CST and RST, respectively), are selected using its corresponding masking vector.

The expected finger output, ${EFO}_{k;k=1:4}$, is derived from the movement task inputs, ${CMD}_{i}$, and the force parameter, FRC. For this purpose, we define the scaled command, $s{CMD}_{i;i=1:2}\{1\to 1;-1\to 0\}$ as,

(6) $$s{CMD}_{i,i=1:2}=\left(1+{CMD}_{i;i=1:2}\right)/2,$$

and enslaving command, $e{CMD}_{i;i=1:2}\{\left(1,0\right)\to \left(0,1\right);\;(0,1)\to (1,0);(1,1)\to (0,0);(0,0)\to (0,0)\}$ as

(7) $$e{CMD}_{i;i=1:2}=max\left(s{CMD}_{i;i=1:2}\right)-s{CMD}_{i;i=1:2}.$$

In this way, we ensure that the enslaving has no effect in the case of the instructed finger, and FRC is irrelevant in the case of the uninstructed finger.

Note, we define the enslaving function (i.e. $0.06\times FRC\times e{CMD}_{i}+0.02\times e{CMD}_{i}$) as a linear equation within the calculations of expected outputs ${EO}_{i}$:

(8) $$\begin{array}{l} {EO}_{i;i=1:2}=\left(FRC\times s{CMD}_{i}+0.06\times FRC\right.\\ \;{\left.\times \;e{CMD}_{i}+0.02\times e{CMD}_{i}\right)}_{i=1:2}. \end{array}$$

Thus, pre-processing calculations of expected finger output yield:

(9) $${EFO}_{k;k=1:4}=\left[{EO}_{1},{EO}_{1},{EO}_{2},{EO}_{2}\right].$$

The error function for each finger output tract k,

(10) $$E_{k;k=1:4}=-{\left({EFO}_{k}-{FO}_{k}\right)}_{k=1:4},$$

is defined as the difference between the expected finger output ${EFO}_{k;k=1:4}$ (equation (9)) and actual output ${FO}_{k}$ for each finger separately. With backpropagation iterations, using a gradient descent technique, the optimal weights to minimize the error function (equation (10)) are determined. First, the derivative for the k output layer outputs of the sigmoid function (equation (1)) is calculated:

(11) $$\partial O_{k;k=1:4}={\left({FO}_{k}\times \left(1-{FO}_{k}\right)\right)}_{k=1:4}.$$

The output delta error for each finger output k is:

(12) $$\delta O_{k;k=1:4}={\left(E_{k}\times \partial O_{k}\right)}_{k=1:4}.$$

The hidden to outputs weights delta correction is:

(13) $${\delta {WO}^{T}}_{kj;k=1:4,j=1:400}={\left({\delta O^{T}}_{k}\times {HO}_{j}\right)}_{k=1:4;j=1:400}.\;$$

Then, weights associated with each finger output k of the connections j between the hidden and output layers are calculated backwards using the delta correction, as follow:

(14) $$\begin{array}{l} {WO}_{jk;j=1:400,k=1:4}\\ \;={\left({FM}_{jk}\times \left({WO}_{jk}^{\text{old}\;}-\eta \times \delta {WO}_{jk}\right)\right)}_{j=1:400,k=1:4}. \end{array}$$

The output delta error (equation (10)) is also backward propagated to the derivative of the hidden layer neurons’ sigmoid function (equation (1)), and results in:

(15) $$\partial {HO}_{j;j=1:400}={\left({HO}_{j}\times \left(1-{HO}_{j}\right)\right)}_{j=1:400}.$$

The hidden delta error for each hidden neuron j is:

(16) $$\delta {HO}_{j;j=1:400}={\left({\left(\delta O_{k}\times {{WO}^{T}}_{kj}\right)}_{k=1:4}\times \partial {HO}_{j}\right)}_{j=1:400}.$$

And the inputs to hidden weights delta corrections,

(17) $$\delta {WH}_{ij;i=1:2,j=1:400}={\left({{CMD}^{T}}_{i}\times \delta {HO}_{j}\right)}_{i=1:2;j=1:400},$$

are calculated and applied to update the input to hidden layer weights ${WH}_{j}$:

(18) $$\begin{array}{l} H_{ij;i=1:2,j=1:400}\\ \;={\left({IM}_{ij}\times \left({WH}_{ij}^{old}-\eta \times \delta {WH}_{ij}\right)\right)}_{i=1:2,j=1:400}. \end{array}$$

To assess the performance of the model, we assessed dexterity with respect to finger individuation and strength for each simulation. In order to calculate the individuation index I, the weighted contributions of the forces from the CST and RST of each finger are first used to calculate fine motor forces (FineMotor1 and FineMotor2 for fingers 1 and 2, respectively), as follows:

(19) $$\text{FineMotor1}=0.75\;*\;{FO}_{1}+0.25\;*\;{FO}_{2},$$

(20) $$\text{FineMotor2}=0.75\;*\;{FO}_{3}+0.25\;*\;{FO}_{4}.$$

That is, the fine motor force for each finger is calculated as 75% of the CST output and 25% of the RST output. The individuation index I between the two fingers is then defined as the absolute value of the ratio of the difference between the fine motor force outputs to their sum [18]:

(21) $$\begin{array}{l} I=|\left({\text{FineMotor}}_{1}-{\text{FineMotor}}_{2}\right)\\ \;\left({\text{FineMotor}}_{1}+{\text{FineMotor}}_{2}\right)|. \end{array}$$

In order to calculate the gross force for each finger (Force1 and Force2), we use different weighted contributions of the CST and RST outputs as follows:

(22) $$\text{Force1}=0.25\;*\;{FO}_{1}+0.75\;*\;{FO}_{2},$$

(23) $$\text{Force2}=0.25\;*\;{FO}_{3}+0.75\;*\;{FO}_{4}.$$

#### 2.2.2. Initialization and parameter setting

We set the global parameters to configure the main structure of the NN, e.g. the number of hidden layer neurons (N=400), number of inputs (NI=2), and number of outputs (NO=4). We defined the dependent parameters to configure the inner structure of the neural network, including the distribution of neurons in the hidden layer. The neurons are divided as follows: 40% are focal CS neurons, consisting of 20% excitatory and 20% inhibitory neurons. Additionally, 20% of the total neurons are shared (i.e. global) CS excitatory neurons, while the remaining 40% are RS excitatory neurons. We used the training and simulation related parameters to control the learning process, error correction resolution steps η=0.01, training cycles as number of days (*n*Days = 360), and training repetition in each training cycle as daily dosage ([50, …, 50, 200, …, 200, 50, …, 50, 0, …, 0] vector of dosage values per day), defined differently for every stage of the training.

For setting up the NN structure we used mask and status variables, ${IM}_{ij}$, ${FM}_{jk}$, and ${NS}_{j}$, respectively (see section 2.2.1, Mathematical Definition), based on the functions’ connectivity between the layers and pre/post stroke status as described in section 2.1 (Model Description and figure 1, ANN Architecture Diagram).

The initial weights of the NN were normally distributed numbers generated from the open interval $\left(0,\;1\right)\left(\sim normal\left(\mu =0.5,\;\sigma^{2}=1/12\right)\right)$ that were then masked using the mask variables, ${IM}_{ij}$ and ${FM}_{jk}$, while the non-connected weights were eliminated (set to ‘0’).

The bias constants were generated similarly to the NN weights to provide additional differentiation among different neurons’ activation functions. They were added as additional parameter inputs to the neurons of the hidden and output layers. These biases were used to compensate for the nature of the input values from one layer to another and the dependencies between the excitatory and inhibitory clusters, and adjust the distribution of the summed values within the desired range of the activation function for a better fitting. The bias setting process required several trial-and-error tests before selecting optimal values for the NN model best fit. The hidden layer bias was set to ‘−6’ and the output layer bias was set to ‘−1’.

#### 2.2.3. Training and simulation methods

For training the ANN, not to be confused with, and not meant to simulate human motor training, the commands of the two fingers (CMD_1_*,* CMD_2_) are fed as inputs to the ANN. The performance of the fingers, i.e. the outputs of the ANN (FO_1_*,* FO_2_*,* FO_3_*,* FO_4_), are calculated in a feedforward propagation. The error function is calculated and minimized in every iteration of the training process using gradient descent in a backpropagation flow (see section 2.2.1, Mathematical Definition). The training for the initial normal condition is achieved by applying a multiday and recurrent dosage force-based motor task to the ‘normal’ (i.e. healthy) pre-stroke ANN, starting from an initially randomized state and converging to the desired instructed finger command behavior. The simulation data is collected throughout the training process for later post-processing and demonstration.

We simulated a stroke by disabling a portion of the neurons in the hidden layer of the trained ANN, simulating lesion overlap in the CS region, as well as RS deficit [34], in proportion to the severity of the stroke, using the *NS* variable (see section 2.2.1, Mathematical Definition). Each cluster of neurons is affected to the same extent in this case, depending on stroke severity. To emphasize this, the lesioned state is reflected by the actual outcome of the injured trained model without further training. In addition to simulating a combined stroke affecting both CS and RS neurons, we also simulated stroke in the CS or RS regions separately by disabling a portion of the neurons in only the CS region or only the RS region. The force-based motor commands are simulated at the stroke condition and the fingers’ outcome values are collected. In conjunction with the stroke, we reduce the learning capability (*η*) in accordance with the lesion severity. As assumed, injured brain plasticity is affected, and hence motor learning ability might be reduced.

Immediately after the lesion, the recovery process of the residual ANN represents the recovery that typically occurs at the early post-stroke phase and may continue up until the chronic phase. The ANN is trained following the same method applied during the initial stage, and the simulation data is collected as well, but with the stroke condition as a starting point.

Since we use all variants of movement commands for training, no additional validation is required for testing the converged NN; however, the quality of the NN convergence is highly dependent on initial values of the weights and, in some cases, needed several trials to reach the optimal NN for the various simulation applications. Configuring different ANN setups is done by setting new values for the ANN global parameters. In addition, the training could be tuned with number of days of recurrent loops with configured dosage iterations and learning factor.

### 2.3. Statistical analysis

Statistical comparison between synaptic weights of the residual hidden layer neurons before and after stroke was conducted using a paired two-tailed *t-test*. Specifically, we compared how the different weights were conditioned when re-trained after stroke. Significance level for all tests was set at 0.05.

## 3. Results

### 3.1. Finger strength and individuation in normal, lesioned and recovered condition

**Figure 2.**
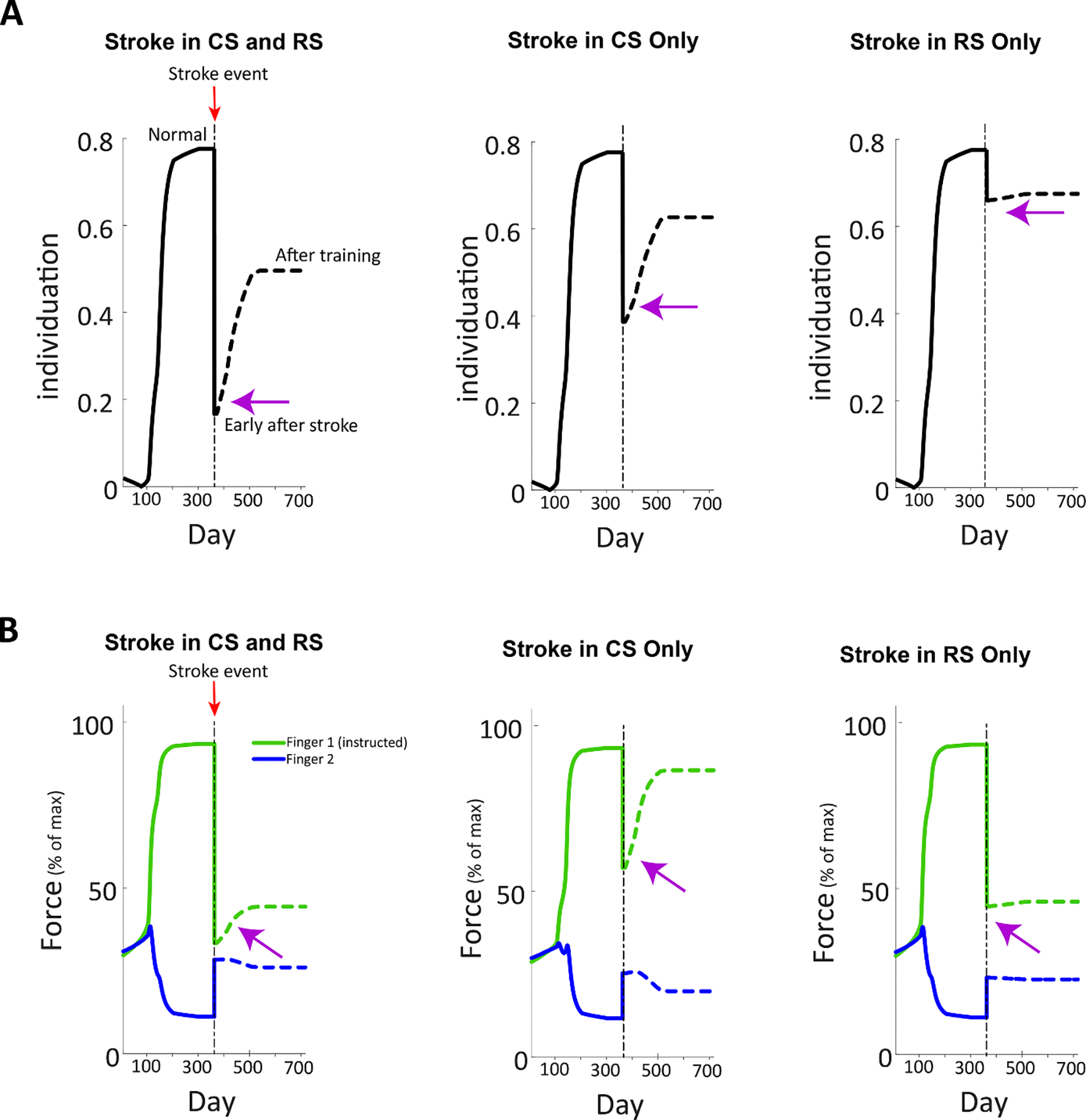
Finger individuation and strength before and after stroke in the CS and RS regions only, CS region only, and RS region only, as predicted by the model. (A). Finger individuation for pre-stroke phase training, in which maximum individuation was achieved, at applied stroke event (50% stroke for each condition), showing individuation reduction between the two fingers, and recovery early after stroke, demonstrating partial recovery of individuation. (B). Finger forces of Finger 1 (instructed) and Finger 2 (uninstructed) during pre-stroke phase training, in which max instructed force and min instructed force are achieved, at application of stroke event, showing lesion acute phase degradation in instructed force and increase in uninstructed/unintentional force, and recovery early after stroke showing enhancement in instructed and uninstructed force behavior. The purple arrow indicates the immediate effect of the stroke on behavior. Command Simulation for all conditions: Finger 1 instructed, Finger 2 uninstructed, Force 100%, Stroke 50%.

The ANN model was first initialized to some intermediate force, starting with randomly generated weights, and then trained for two fingers (Finger 1 and Finger 2) to a pre-stroke condition by applying the set of commands alternately to each finger in the same training set. At the end of this initialization process, the ANN was fully capable of the trained motor functionality of the two fingers for the selected commands. The simulation data was collected throughout the training convergence and demonstrated the model behavior of this stage. The simulation showed the individuation between the instructed/uninstructed fingers (i.e. maximizing the instructed force and minimizing the uninstructed force) and the enhancement in the strength of the fingers (see figures 2(A) and (B), respectively) for post 50% stroke in the CS and RS regions, CS region only, and RS region only. Prior to stroke, the instructed force reached 93.04%, 93.25%, and 93.4% of the maximum force for Finger 1 in the CS and RS regions, CS region only, and RS region only conditions, respectively. For these same conditions, the uninstructed involuntary force reached 11.15%, 11.76%, and 11.14% of the maximum force, respectively, for Finger 2. Before application of stroke, the individuation between Finger 1 and Finger 2 for all three conditions was measured as 0.78.

A stroke event was applied by deleting 50% of the neural network from the hidden layers, simulating 50% lesion load in the CS region and 50% neuronal deficit in the RS region, 50% lesion load in the CS region only, and 50% deficit in the RS region only, for the CS and RS regions, CS region only, and RS region only conditions, respectively. The three simulations were executed at this point and exhibit the behavior of the impaired model via a drop in the instructed force, rise in the uninstructed force and detriment to the individuation between the two fingers (see figure 2). The instructed force of Finger 1 reached 33.37%, 57.15%, and 44.6% of the maximum force for the three conditions, respectively, while the uninstructed force of Finger 2 reached 28.29%, 25.26%, and 23.12% of the maximum force for the three conditions, respectively. The individuation was calculated as 0.17, and 0.39, and 0.66, respectively.

Following the stroke event, i.e. proceeding from the stroke condition state, the model was trained using the same training method to regain some enhancement of the motor behavior and to represent the recovery early after stroke. Similar to the pre-stroke stage, simulation data was captured throughout the training process up to the limit of the impaired ANN convergence. We observed a rise in the instructed force and drop in the uninstructed force, leading to enhancement in the individuation (see figure 2, post-stroke recovery). The instructed force of Finger 1 reached 44.33%, 86.53%, and 46.03% of the maximum force for the three conditions (i.e. CS and RS regions, CS region only, and RS region only), respectively, while the uninstructed force of Finger 2 reached 26.02%, 19.85%, and 22.58% of the maximum force for the three conditions, respectively. The individuation was calculated as 0.5, 0.63, and 0.67, respectively.

In comparing between the three stroke conditions, it can be seen that finger individuation was most damaged by stroke in the CS and RS regions, followed by the CS region only, and then the RS region only. Finger forces, again, were most impaired by stroke in the CS and RS regions. However, here, RS-only stroke shows worse force profiles than CS-only, exhibiting greater reduction in instructed finger force. Enslaving in the CS-only stroke was slightly worse than RS-only stroke in the acute post-stroke phase.

### 3.2. Increased activation of uninstructed finger as a function of instructed finger strength

**Figure 3.**
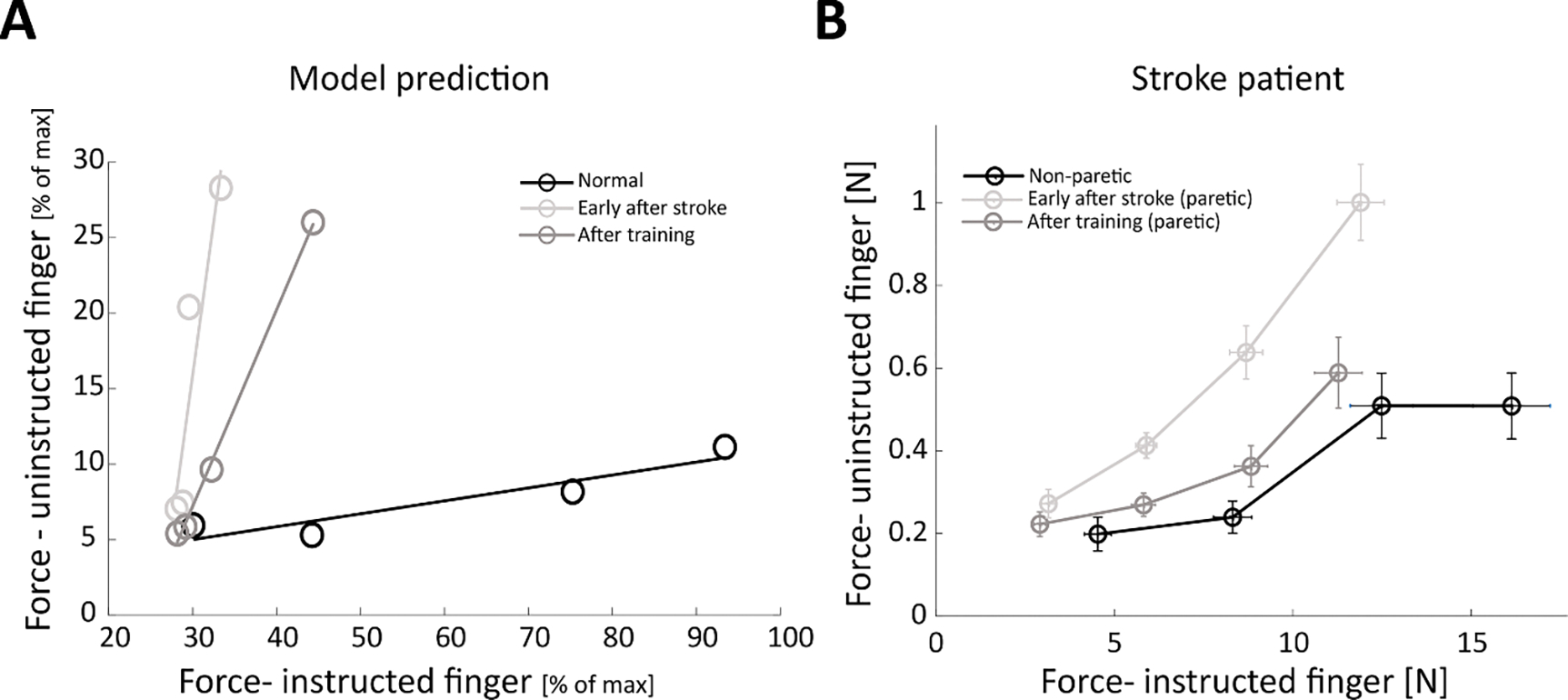
Uninstructed forces as a function of instructed finger strength. Model prediction (left) vs. clinical measurements (right): Recovery effect on finger individuation as predicted by the model and as observed in a stroke patient. (A). Forces in the uninstructed finger plotted against the force generated by the instructed finger at multiple force amplitudes as predicted by the model. Lines represent normal pre-stroke phase (black), acute phase (light grey), post-recovery (induced spontaneously or with training early after stroke) and chronic phase (dark grey). Model parameters: (Finger 1 instructed, Finger 2 uninstructed, Stroke: 50%, Forces: 40%, 50%, 75%, 100%). (B). Reduced enslaving in the individuation task in a stroke patient (data from [7]). Forces of the non-instructed finger as a function of the forces in the instructed finger for the non-paretic hand reflecting the pre-stroke baseline level (black), early after stroke (light grey) and after training (dark grey). Note that while force was measured in N for the stroke patient (B), force is represented as % of max for the model (A). Maximum force for the instructed and uninstructed finger is not necessarily equivalent and thus comparison between A and B of proportion of uninstructed to instructed force should not be made, but rather only between the general pattern of behavior.

Next, we sought to explore the relationship between the force of the uninstructed finger for different force levels of the instructed finger. To test this relationship in our model, we repeated the simulations with different force targets and measured the involuntary uninstructed force from Finger 2 and the instructed force from Finger 1 in the different phases (pre-stroke, early after stroke, and after training) of these simulations for each force target. In figure 3(A), we plotted uninstructed vs. instructed forces (normalized to max force) of our simulation, and compared them to similar clinical results, in figure 3(B), of a stroke patient from Mawase *et al* [7] (measured in Newtons). The *y*-axis represents the uninstructed finger force (i.e. involuntary force) corresponding to the applied force of the instructed finger as shown on the *x*-axis. The slope ratio represents the individuation ability of the instructed finger. We see that early after stroke, the uninstructed finger force increased (i.e. reduced individuation) to more than it was in the normal/non-paretic case in both the model and stroke patient graphs.

As expected, after training (e.g. induced by spontaneous recovery and/or additional rehabilitative training), we observed that the model predicted reduced involuntary uninstructed force (i.e. increased individuation) that was closer to a normal level (figure 3(A), Model Prediction). Quantitively, this reduction was captured by the slope of a linear regression line that was fitted to each data set and showed an almost flat line (slope = 0.12, with 95% CI of 0.085–0.15) in the normal condition (i.e. before stroke), substantial increase in slope (slope = 0.54, with 95% CI of 0.05–1.03) immediately after stroke and reduction (slope = 0.39, with 95% CI of 0.06–0.71) after recovery. This instructed-uninstructed finger force relationship replicated what we have previously reported in human stroke patients (figure 3(B), Stroke Patient: shows actual data from a stroke participant during an individuation task [7]). Note that in figure 3 we compare the non-paretic hand of the stroke patient to the normal condition of the model. While the non-paretic hand may be affected to some extent by stroke, we cannot obtain ‘normal condition’ data from stroke patients, and thus the non-paretic hand served as the closest approximation to the normal condition.

### 3.3. Effect of CS-lesion overlap and RS deficit on finger individuation

**Figure 4.**
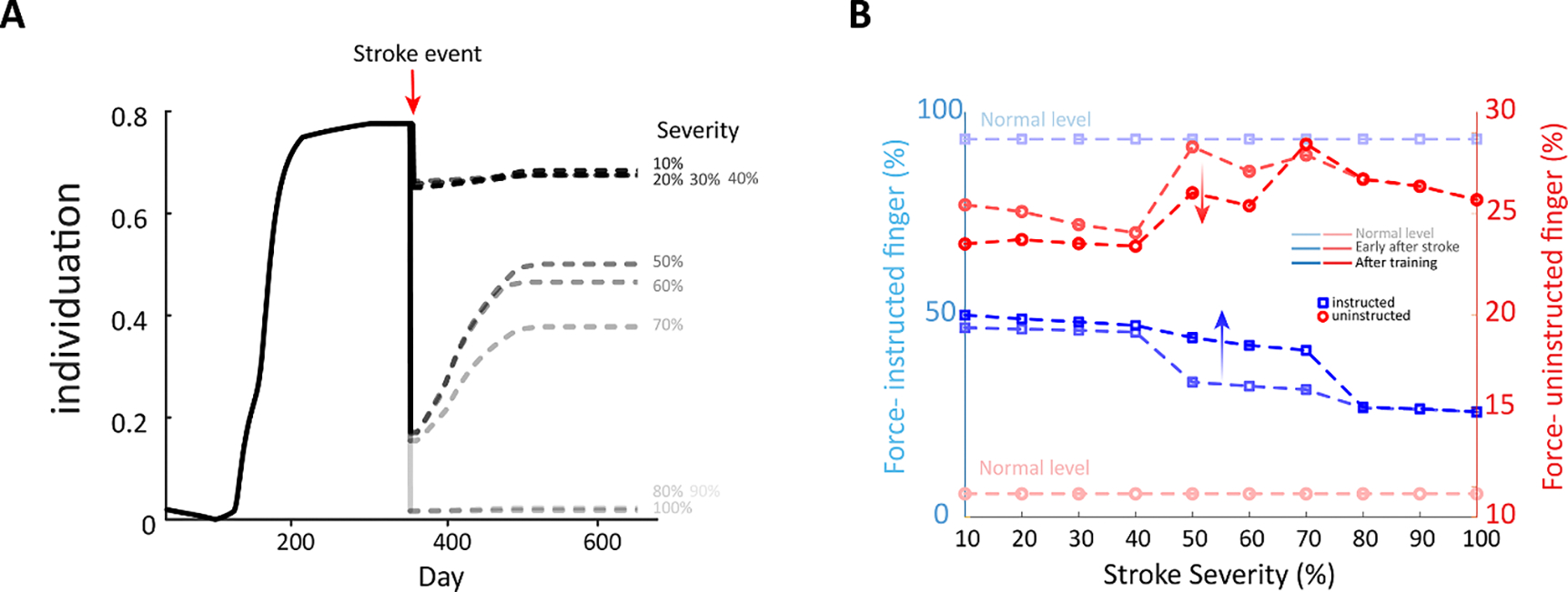
Effect of stroke severity on instructed vs uninstructed fingers. Model parameters: (Stroke 10%–100%, Force 100%). Simulations: All are trained similarly during pre-stroke phase, then stroke with different severity (simulating % CS overlap and % RS neural deficit) is applied. We recorded the results early after stroke at sub-acute phase, then during recovery in chronic phase and at the end of rehabilitation effort. (A). Individuation between instructed and uninstructed fingers in accordance with stroke severity. The graph demonstrates that the greater the stroke severity, the more severe the stroke effect on individuation, and the less likely recovery is after stroke. Stroke severity ⩽ 40% has relatively minimal effect on the individuation and recovery. (B). The dynamics of finger strength following lesion with different percent stroke severity. In the instructed finger (blue lines), the greater the stroke severity, the less instructed force that can be produced by the model, starting with the light blue capturing the forces early after stroke, the darker blue during recovery and darkest blue at end of rehabilitation process. Recovery increases from the acute post-training phase, i.e. increase in instructed force. The uninstructed force (light red lines) is inversely affected by the stroke severity early after stroke and during recovery. After training, however, the uninstructed force (dark red) is directly affected by stroke severity, as seen by reduced uninstructed force levels compared to what is predicted early after stroke.

We measured the effect of stroke severity on finger individuation during the acute phase and after training (figure 4(A)). We then tested a resulting assumption that effect of CS-RS lesion load on finger individuation was driven by the relative changes between instructed and uninstructed fingers. To test this, we measured the effect of stroke severity, as predicted by the model, on the instructed and uninstructed forces early after stroke and after recovery completion (i.e. after training). We plotted the uninstructed and instructed forces vs. stroke severity on the same plot for comparison (figure 4(B)). A large CS-lesion load in our model was presumed to reflect a large infarct in the cortical CS region. Our model predicted that small to moderate lesion overlap (i.e. up to 10%–40% of the total neurons in the hidden layer CS and RS regions) results in relatively small degradation in individuation (figure 4(A)), and less degradation in instructed and uninstructed forces compared to larger lesion overlaps (figure 4(B)). In moderate to large strokes (i.e. 50%–70% of hidden layer CS and RS neurons), we see a quite drastic negative effect of the stroke on individuation in the acute phase, with substantial but incomplete recovery after the training process. Detriment to instructed and uninstructed forces in the acute phase was more pronounced in this range of lesion overlap, with noticeable but incomplete recovery following training that asymptotes to levels well below full recovery. These below-normal-function levels were related to the severity of the stroke and the amount of training induced by the model. Finally, the model predicted that severe lesion in the range of 80%–90% caused a nearly complete drop in finger individuation that cannot be effectively restored, and additional training enhancements were nonexistent. With respect to finger forces, there was a detriment to uninstructed finger forces (enslaving) at the severe level that compares to the higher end of the moderate stroke severity level, and detriment to instructed finger force that was greater than all other stroke severity levels. Here too, training did not lead to visible recovery.

### 3.4. Changes in synaptic weights in residual CS and RS during stroke recovery

**Figure 5.**
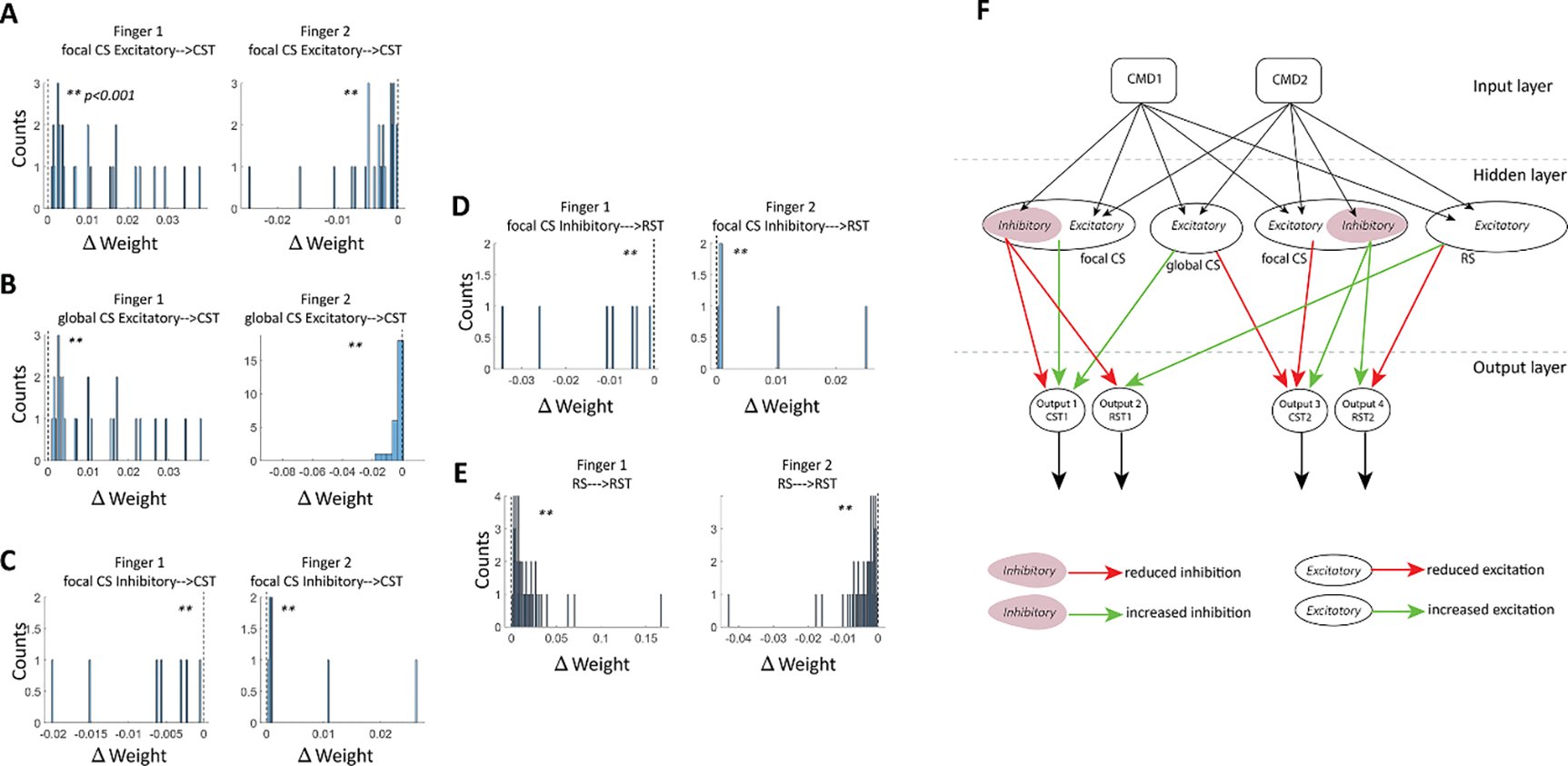
Weight Gain Histograms for Model Simulation. Model Simulation Parameters: (Finger 1 instructed, Finger 2 uninstructed, Stroke 65%, Force 100%). (A). Weights of focal CS excitatory neurons to CST output increased for Finger 1 and decreased for Finger 2. (B). Weights of global CS excitatory neurons to CST output increased for Finger 1 and decreased for Finger 2. (C). Weights of focal CS inhibitory to CST output decreased for Finger 1 and increased for Finger 2. (D). Weights of focal CS inhibitory to RST output decreased for Finger 1 and increased for Finger 2. (E). Weights of RS neurons (global excitatory) to RST output increased for Finger 1 and decreased for Finger 2. (F). Simplified representation of the network architecture derived from detailed architecture in figure 1; red arrows—reduction in weights, green arrows—increase in weights. Abbreviations: CMD—command, CS—corticospinal, RS—reticulospinal. ** indicates *p* < 0.001.

The observation of improved finger individuation after stroke is classically believed to be associated with plasticity changes in network connectivity of the residual neurons. Here, we investigated how the connectivity strength of the model changed during the recovery process, i.e. how the different weights were conditioned when re-trained after stroke. We evaluated a representative case, 100% force and 65% stroke severity, which demonstrated the recovery enhancements in both force and individuation of the two fingers (figure 5).

We found increased plasticity of the residual CS and RS neurons, as reflected by weight gain increase or decrease from the hidden layer neurons to the finger outputs of the instructed and uninstructed fingers (paired *t-test; p* < 0.001 for all results). Specifically, for Finger 1 (instructed), weight gains increased for all excitatory neurons to their associated outputs (i.e. focal and global CS excitatory neurons to Output1, and RS neurons to Ouput2 of Finger 1; see left graphs in figures 5(A), (B), (E) and (F)). This was accompanied by a decrease in weight gains in all inhibitory neurons to their associated outputs (i.e. focal CS inhibitory neurons to Output1 and Output 2 of Finger 1; see left graphs in figures 5(C), (D) and (F)). For Finger 2 (uninstructed), weight gains decreased for all excitatory neurons to their associated outputs (i.e. focal and global CS excitatory neurons to Output1, and RS neurons to Ouput2 of Finger 2; see right graphs in figures 5(A), (B), (E) and (F)). This was accompanied by an increase for all inhibitory neurons to their associated outputs (i.e. focal CS inhibitory neurons to Output1 and Output 2 of Finger 2; see right graphs in figures 5(C), (D) and (F)). In summary, improved finger individuation was achieved by strengthened connectivity of all excitatory neurons, and weakened connectivity of all inhibitory neurons, projecting to both outputs of the instructed finger, as well as weakened connectivity of all excitatory neurons, and strengthened connectivity of all inhibitory neurons, projecting to both outputs of the uninstructed finger.

### 3.5. Robustness analysis

**Figure 6.**
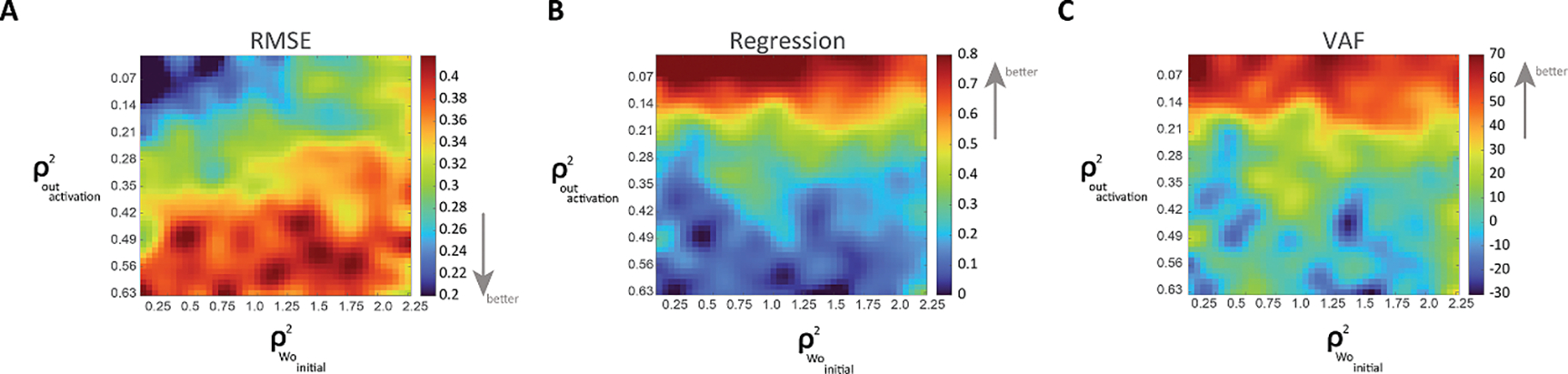
Robustness testing of the ANN model of finger individuation. Noise was injected into the system in two different places: (1) the initial values of WO (weights of the hidden-to-output layer), where the noise was sampled from a normal distribution with variance ranging from 0.01 to 0.675, and (2) in the output activation function (output sigmoid function), where the noise was sampled from a normal distribution with variance ranging from 0.05 to 2.25. A. Root Mean Square Error (RMSE) between the model output and a reference output, using a 65% stroke in both CS and RS neurons. B. Regression coefficient between the model output and the reference outcome as a function of noise level. C. Variance Accounted For (VAF) metric, showing the proportion of variance in the reference outcome explained by the model output across different noise levels.

In order to test the robustness of our network, we conducted a comprehensive analysis to assess the stability and reliability of our neural network model under varying conditions. Specifically, we artificially injected noise (normally distributed) with variable standard deviations at two points in the model: (1) Initial values of weight links WO (see section 2.2.1, Mathematical Definition); We applied noise ([0.05–2.25]) to the weight links WO. The starting conditions were drawn from a diverse pool of values, represented on the *x*-axis in figure 6. (2) Hidden output activation function; We also introduced noise ([0.01–0.675]) in the calculation of the hidden output activation function, shown on the *y*-axis in figure 6. To evaluate the impact of these variations, we compared the results against a reference model representing a 65% stroke in both the CS and RS regions. Our comparison metrics included the Root Mean Square Error (RMSE; see figure 6(A)), regression coefficients (see figure 6(B)), and the explained variation (VAF; see figure 6(C)) between the model’s outputs and the reference data.

Our analysis revealed that the error (RMSE) remained relatively low and within acceptable limits when the variance of noise in the hidden output function was below ~0.21 (*y*-axis) and when the variance of the initial weight WO was below ~1.25 (*x*-axis). This indicates that the model’s predictions are robust at low noise levels in both the hidden output activation function and the initial weight of the hidden-to-output layer. The regression coefficients and VAF showed consistent patterns but were less sensitive to noise in the initial weight WO, suggesting that the model primarily maintains its predictive accuracy in the hidden output function. These results highlight the model’s robustness to internal noise, reliably producing outputs that align with expected physiological behavior in stroke-affected CS and RS systems. This robustness ensures that the model’s predictions are not overly sensitive to specific assumptions or parameter settings, enhancing its applicability to real-world physiological conditions.

## 4. Discussion

In the present study, we designed an ANN model with physiologically-based architecture that models recovery of finger dexterity after stroke. Our central result is that an ANN trained to produce finger individuation exhibited dynamics that strongly resemble that of healthy individuals and patients after having recovered from a stroke. The resemblance between the model outcome and reported data in previous clinical works was manifested by the substantial reduction of finger individuation immediately after stroke [2, 7], the recovery pattern following training, the near-linear relationship between uninstructed and instructed finger forces and the relationship between size of CS-RS-lesion overlap and severity of impairment in finger individuation [5–7]. Notably, we did not achieve this by fitting the ANN to actual clinical data. Rather, the agreement between model outcome and clinical data emerged as a result of the architecture of the excitatory/inhibitory cortical CS and subcortical RS neuron pools needed to generate the normal patterns of individuation. Importantly, once initialized to pre-stroke condition, our solution is capable of simulating dynamic functional capacity of the cortical motoneurons throughout the lesion event and the recovery process that follows. In addition, the model makes predictions that might provide mechanistic explanation about the functional reorganization of the cortical and subcortical network during recovery of control of finger movement.

Our modeling study provides a framework by which to understand a number of experimental findings related to finger dexterity. First, the pattern of instructed forces, uninstructed forces and individuation in a normal condition, as seen in figure 2, mimics the convergence relation between the instructed to uninstructed force levels as observed in humans and primates [8, 10]. Second, the immediate ANN response to the simulated stroke event in the CS and RS regions revealed increased involuntary uninstructed forces that were driven by weakening of the instructed finger and exaggerated force of the uninstructed finger. This is in agreement with documented clinical observations of acute poststroke phase finger functionality in which post-stroke patients exhibit reduced instructed finger force capacity and increased uninstructed forces [3, 35]. Third, the model exhibited how the impaired motor system re-adjusted and learned new neural activation of the residual cells to compensate for the loss in finger control during the recovery process. Behaviorally, we observed an increase in the instructed force and decrease in the uninstructed force, and eventually enhancement in finger individuation during the recovery period. This is in line with recent studies that showed meaningful improved, yet incomplete, finger individuation during the early months after the stroke event [5, 22]. We were also able to simulate strokes in the CS region only and in the RS region only. Our findings further suggest the existence of a neural circuit with interactive dynamics between the CS and RS regions that processes finger commands to produce the necessary motor outputs, such as force and/or individuated finger movements. Notably, our model’s results align with the seminal works of Lawrence and Kuypers (1968) [8, 16], demonstrating that lesions in the CST dramatically impair finger individuation, while lesions in the RST primarily affect finger strength and, to a lesser extent, individuation. Furthermore, recovery from stroke-related finger control deficits involves changes in the neurons within the residual CST and RST, as previously observed in monkey studies by Zaaimi *et al* [15].

It is of special note that our general model architecture is based on that of Norman *et al* [18] (which is itself based on the model of Reinkensmeyer *et al* [36] that simulates wrist flexion and extension following stroke). However, there are numerous important differences. (1) Norman *et al* do not describe an input layer, and they have a single finger individuation task in which the objective is to perform maximum force with the instructed finger and minimize force from the uninstructed finger. They do not support variable instructed force values, and the resulting output forces are determined wholly by the weights and a saturation function, and are updated until the maximal/minimal forces are achieved. We, on the other hand, describe an input layer in which the finger individuation task is determined by the two finger inputs (specifying which finger is instructed) and the encoded force that can be any instructed force in the range of (0,100%], representing the whole human range of possible dexterous individuation actions (see section 2.1.1, Input Layer). The encoded force has a direct impact on the input to the hidden layer CS and RS neurons, along with the weights, which are updated until the output force is as close as possible to the instructed force, and the uninstructed force is minimized. This being the case, we were able to simulate the relationship between instructed and uninstructed forces throughout the whole range. (2) We use backpropagation for our learning algorithm instead of reinforcement learning. Backpropagation has also been suggested to employ biologically plausible features and is the most common type of learning used in these types of neural network applications, shown in general to better represent observed neural responses than many other learning models [37]. (3) Importantly, we simulate a single network in different conditions, rather than two separate lesioned and non-lesioned networks, allowing us to simulate a more ‘naturalistic’ event of stroke and recovery. We use this single network over ‘natural’ time which first undergoes learning to reach the normal healthy condition, then undergoes neuronal death to simulate the stroke condition, and finally undergoes recovery and neurorehabilitative training. In contrast, Norman *et al* used two separate networks, and the lesioned network was not the same network that was already trained to the healthy condition. (4) A major advancement over the previous Norman *et al* model is the ability of the present model to simulate stroke in multiple locations (i.e. CS and RS regions, CS region only, and RS region only). This approach makes our model more modular and physiological and provides an important basis for adding more complexities in future study.

### 4.1. Cortical-subcortical neural basis of finger individuation

Our model predicts post-lesion plasticity in both cortical and subcortical areas for recovery of finger dexterity. While strengthening of the connectivity in the residual descending cortical pathway seems to contribute to a larger extent to fractionating finger movement, strengthening of the RST seems to compensate for the loss of finger strength [15]. Inspection of changes in synaptic weights during stroke recovery as predicted by the model revealed plasticity in the residual CS and RS. Specifically, we observed strengthening of the weights of the focal and global cortical excitatory CS neurons to the instructed finger CST and RST outputs, as well as global excitatory RS neurons to the RST output. Strengthening of the weights to the CST output indicates enhancing the individuation and, to some extent, the force of the instructed finger. Additionally, strengthening the weights between the RS neurons, that serve for applying force, and RST output has a faciliatory effect on the strength of the instructed finger. On the other hand, we reported weakening of the weights that connected the focal inhibitory CS neurons to both the CST and RST outputs of the instructed finger. This reduced contribution inversely affected the individuation and force of the instructed finger, and thus weakening them is in favor of individuation. With respect to the uninstructed finger, we observed weakening of the focal and global cortical excitatory neurons to the uninstructed finger CST and RST outputs, as well as global excitatory RS neurons to the RST output. Weakening of the excitatory neurons may facilitate minimization of enslaving of the uninstructed finger by reducing excitation to the finger muscles. We also reported strengthening of the weights that connected focal inhibitory CS neurons to both the CST and RST outputs of the uninstructed finger, which may further improve individuation by inhibiting the uninstructed finger muscles.

There have been several studies investigating the residual neurons in the primary motor cortex (M1) and their role in recovery after a cortical lesion. These studies often focus on the mechanisms of plasticity, reorganization, and the contribution of surviving neurons to motor recovery in animal models. For example, Nudo and Milliken [38] demonstrated that after a focal ischemic infarct in the primary motor cortex of adult squirrel monkeys, there was significant reorganization of the remaining cortical areas. Intensive rehabilitation led to the expansion of the hand representation into adjacent cortical areas, indicating that plasticity in the residual neurons can support functional recovery. Castro-Alamancos and Borrel [39] also showed that surviving neurons in the M1 of rats exhibit increased excitability and synaptic strength after a lesion. These changes were correlated with improved motor function during recovery, suggesting that residual neurons adapt to compensate for the lost function. As for the role of the residual neurons in the RS and their role in recovery after a cortical lesion, the study by Zaaimi *et al* [15] indicated changes in the connectivity of descending motor pathways, including those from residual neurons in the RS, after a CS lesion. These changes suggested that RS systems might sub-serve some of the functional recovery after CS lesions.

To the best of our knowledge, no studies have specifically examined changes in inhibitory cortical neurons during the recovery process related to fine-motor control of fingers. This area remains essential for future research. A recent study by Griffin and Strick [24] demonstrated that cells in the primary motor cortex (M1) can control the inhibition of antagonist muscles in wrist movements, doing so a few milliseconds before activating the agonist muscles. Whether this mechanism applies to finger individuation remains to be determined in future investigations.

### 4.2. Severity of impairment in finger dexterity correlated with size of CS-lesion overlap in the motor cortex and motor-related subcortical areas

There is mounting evidence suggesting that lesion load within specific brain areas might be a major factor in the ability to restore motor function after stroke, and the improvement, or lack thereof, in motor activity [40–42]. Several studies have demonstrated that greater damage to the CS projections is associated with more impairment in stroke patients [43–45]. Our model demonstrates the correlation between lesion load in the CS and RS region and reduced plasticity of the injured brain, which explains the (in)ability to restore motor function in these cases (figure 4). In our model, a small to moderate lesion load around 10%–40% induces less impairment in the motor function compared to greater loads. This might be explained by the fact that the NN model is converged to a ‘mathematically’ stable global minimum and requires a substantial ‘hit’ to be greatly disturbed. This aspect of our model is in accordance with clinical data of stroke, as the effects on function of mild strokes can be difficult to quantitatively assess [46–49]. In the range of 50%–70% lesion load, we can observe a much greater effect of the stroke event in our model. The modeled acute phase emphasizes the level of the disturbed motor function relative to the size of the injured region and shows that there is still room for a certain amount of spontaneous recovery of motor capabilities, but that it is limited inversely to the magnitude of the lesion severity. In our model, lesion load with more than 80% dead neurons demonstrates severe impairment of motor function that is almost incapable of being restored and minimally or not responsive to rehabilitation. The clinical analogue has been documented in the literature with poor functional outcomes for severe stroke and a more difficult challenge to design and implement rehabilitation protocols capable of inducing improvements in this population [50, 51]. Nevertheless, neurophysiological quantification of residual CS neurons that survive after the stroke, as well as association between this quantity and motor impairments, require future research with high-resolution imaging tools.

For 10%–40% stroke we see relatively similar values for instructed and uninstructed force (figure 4(B)). This is in correspondence to the similar pattern of detriment to finger individuation in this stroke range. For 50%–70% stroke we see a trend of increasing uninstructed force and decreasing instructed force with stroke size early after stroke and after training. Interestingly, in the very severe range of stroke (i.e. >70%), despite abnormalities in both instructed and uninstructed force patterns, we observe a trend of decreasing uninstructed force and decreasing instructed force with increasing stroke size during the acute and sub-acute (post-training) stroke phases. A possible explanation for this is that the model’s post-stroke ‘motor system’, including CS and RS, has less available neurons that can affect force, and since the involuntary forces of the uninstructed finger are linearly affected by the amount of applied force in the instructed finger, when less instructed force can be generated by the model’s ‘motor system’, it will lead to less uninstructed force as well.

Since individuation (figure 4(A)) is calculated based on the normalized difference between instructed and uninstructed fine motor output forces (figure 4(B)), we observed that immediately and early after stroke, the magnitude of the decrease in instructed forces across different lesion loads is greater than the magnitude of the increase in uninstructed forces. Consequently, individuation decreases as the CS-RS lesion load increases. Recovery induced by training and/or occurring spontaneously caused increase in the forces of the instructed finger and decrease in the uninstructed forces. This can be explained as enhancement in the focal segments, excitatory and inhibitory weights, with the excitatory neurons positively affecting the instructed forces and, conversely, the inhibitory neurons negatively affecting the uninstructed forces.

Altogether, these results indicate that interactive dynamics between cortical and subcortical neurons could provide a biologically plausible explanation for the recovery of finger dexterity. It is essential to understand which subsystems contribute to recovery of finger movement in order to provide a rational basis to develop circuit-level therapeutic strategies that will optimize rehabilitation. Some aspects of the reported finding are in accordance with well-reported clinical and neurophysiological outcomes, but others provided mechanistic prediction of the interactive relationships in the neural network that underlie finger dexterity. These predictions can be tested in future research working primarily with human and/or animal models that typically exhibit finger dexterity.

### 4.3. Limitations of the study

Our model has some limitations. First, in this work we limited our model to two fingers in order to demonstrate the potential relevance of our model. A full model including the entire hand representing complex multifinger motor behaviors is of ultimate interest, however individuation naturally differs markedly for each finger, or between each combination of fingers in multifinger tasks [52–55]. Further, each of the fingers and each combination of them have different anatomical constraints with respect to individuation and strength which may be due to neural aspects of control for each finger or pattern of fingers, mechanical coupling constraints that rise from the different bony and muscular architecture of each finger, different ligamentous and tendinous connections, etc [56, 57]. Our model can be expanded to include five fingers, however a complex model which truly represents the whole hand must take into account the aforementioned important factors. Our model may provide an important basis for a more complex naturalistic model.

In addition, our model has some versatility with regards to stroke simulations, with the ability to simulate stroke in the CS and RS regions, CS region only, and RS region only. This is a substantial advancement over the previous model of Norman *et al* [18]. However, in the case of stroke in both the CS and RS regions, the stroke was applied to all segments of the NN neurons by deactivating the same percentage of neurons. This was true for all cluster types including CS excitatory and inhibitory neurons, as well as RS neurons. These assumptions do not necessarily represent the real organization of the motor system, nor do they reflect how a real stroke lesion may differently affect motor divisions and representation of multiple fingers. In fact, it was shown that control of individuated finger movement is widely distributed in the primary motor cortex [2], and electrical stimulation often elicited involuntary movements of multiple fingers [10, 58]. Our model in the present study, for the case of stroke in both the CS and RS regions, was intended to simulate recovery from stroke with the residual capacity of both the CS and RS and thus provides an important basis for future study, which may simulate stroke to a different degree in the CS and RS regions. The scenario in our present model does not represent all stroke profiles, however it may be representative of a common profile, for example with lesion around the area of the internal capsule, or in general, subcortical strokes, where the CS and cortico-RS regions are in very close proximity to each other and are likely both affected by stroke [34, 59]. In fact, Karbasforoushan *et al* [34] examined microstructural damage to various sensorimotor pathways, due to unilateral subcortical stroke, using high-resolution structural MRI of the brainstem and spinal cord—areas where the pathways can be differentiated, and indeed found that decrease in white matter integrity of the CST was accompanied by decrease in integrity of the RST as well. Regardless, future work is underway to investigate alternate approaches. But importantly, our results show substantial similarity to clinical works of the same type, and we believe that our model constitutes an important advancement toward prediction of recovery of finger dexterity following stroke.

Finally, our model is an oversimplification of the proposed network that potentially underlies recovery of finger dexterity. Simplification of the proposed ANN model was pronounced in its architecture, including design, connections, and training. Thus, although our model predicated plausible outcomes that highly resembled data from human and/or primate research, it seems that more complex architecture of neural networks including additional brain areas, beyond CS and RS regions, must be involved in control of finger movement. Our present model represents a specific facet of recovery of hand motor function following stroke. It was our focus to describe the specific contributions of the CS and RS regions to recovery, as evidence suggests that these areas are the main contributors to control of finger movement [8, 13, 16, 57], and as such, the major regions involved in the recovery process of finger dexterity[15]. However, for instance, an intriguing but underexplored area that may contribute to finger control is the magnocellular red nucleus in the midbrain and its descending rubrospinal tract (RuST). Many neurons in the magnocellular red nucleus excite the wrist and extrinsic finger muscles of the contralateral upper extremity [60]. However, in humans, the magnocellular red nucleus is relatively small, and the RuST does not extend below the upper cervical segments [61]. Thus, the potential role of the RuST in finger control remains speculative and has not been thoroughly examined during individuated finger movements in humans. Consequently, we have chosen to exclude this area from our model due to the lack of significant findings confirming or ruling out its contribution in humans.

### 4.4. Future directions and summary

As for future directions, though the ANN model was limited to two fingers for our simulation requirements, the general model is easily scalable to support all fingers. In this case, the level of complexity of the model increases, and the training set must be revised accordingly, and thus simulation run time increases substantially. Such enhancements and/or adding further motor-related divisions may be optimally addressed using one of the commonly used advanced computations. More advanced neural network models or deep learning frameworks might be used and trained to simulate enhancements in both strength and individuation in hand motor function, based on existing clinical experiments and available data (e.g. size of lesion and affected part/s of the brain motor divisions).

Our model makes clinically-testable predictions. For example, it predicts that post-stroke CST/RST integrity is correlated with improved finger dexterity recovery. This prediction could be verified in a clinical study wherein CST/RST tractography from diffusion-weighted MRI is used to predict patient outcome. This protocol could validate this model finding and possibly lead to future clinical work that stratifies patients into therapeutic interventions based on CST/RST tractography and the model’s predictions of expected recovery. Thus, ultimately, treatment can be planned based on the desired target goals for finger individuation and/or strength. The improvement, or lack thereof, in the motor activity as predicted by the model will help us estimate the amount of motor recovery of the training dataset. For example, with respect to our model prediction for a patient with a pure cortical lesion with 50% CS region overlap (as could be validated by CT or MRI scans), our recommendation would be harnessing intensive appropriate rehabilitation techniques to allow the residuals of the affected area to recover. In this case, this may include more focus on intensive finger individuation training, rather than strength training. In addition, our model predicts that upregulating the neurons of the RS system would improve recovery of finger dexterity. One approach to do this is by applying brain stimulation techniques targeting cortical origins of the RST, i.e. cerebro-reticular tract originating from premotor areas [62]. Indeed, a new study showed that, in addition to conventional rehabilitation, using invasive deep cerebellar stimulation, that likely affected many brainstem structures including the RS formation, improved clinical outcomes in stroke patients compared to a group that underwent conventional therapy only [63].

## Data Availability

All data that support the findings of this study are included within the article (and any supplementary files).
